# A case of reassortant seasonal influenza A(H1N2) virus, Denmark, April 2019

**DOI:** 10.2807/1560-7917.ES.2019.24.27.1900406

**Published:** 2019-07-04

**Authors:** Ramona Trebbien, Anders Koch, Lene Nielsen, Dår Kristian Kur, Pontus Westerström, Tyra Grove Krause

**Affiliations:** 1 National Influenza Center, Statens Serum Institut, Copenhagen, Denmark; 2 Department of Infectious Disease Epidemiology and Prevention, Statens Serum Institut, Copenhagen, Denmark; 3 Department of Clinical Microbiology, Herlev Hospital, Copenhagen University, Herlev, Denmark; 4 Department of Clinical Biochemistry, North Zealand Hospital, Hillerød, Denmark; 5 Department of Pulmonary and Infectious Diseases, North Zealand Hospital, Hillerød, Denmark; 6 Department of Infectious Diseases, Rigshospitalet University Hospital, Copenhagen, Denmark*

**Keywords:** reassortant, reassortment, influenza, H1N2, seasonal, H1N1, H3N2, surveillance, influenza virus

## Abstract

A reassortant influenza A subtype H1N2 virus with gene segments from seasonal A(H1N1)pdm09 virus (HA, MP, NP, NS, PA, PB1 and PB2) and seasonal A(H3N2) virus (NA) was identified in a routine surveillance sample in Denmark. The patient recovered fully. This is the second reassortant influenza A(H1N2) virus identified in Europe in the 2018/19 influenza season, with the first case being detected December 2018 in Sweden.

A reassortant seasonal influenza A(H1N2) virus was identified in Denmark in a routine surveillance sample from a naturally infected patient who recovered fully. Here we describe the case and virological characterisation of the virus.

## Influenza surveillance in Denmark

National epidemiological and virological surveillance of influenza in Denmark is conducted at Statens Serum Institut (SSI), Copenhagen, Denmark, which harbours the National Influenza Centre (NIC). The NIC receives samples from two sources: (i) the sentinel system, which involves general practitioners submitting samples from patients with influenza-like illness (ILI) and (ii) influenza virus-positive diagnostic samples from regional clinical microbiological departments, a subset of which are included for real-time subtyping at the NIC during the influenza season.

**Figure 1:**
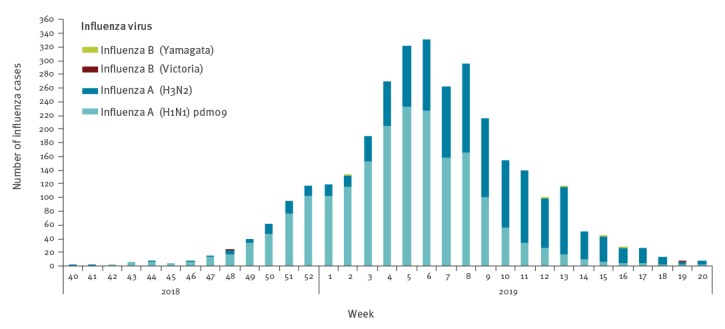
Distribution of circulating influenza virus types and subtypes, Denmark, influenza season 2018/19

Both influenza A(H1N1)pdm09 virus and influenza A(H3N2) virus circulated throughout the 2018/19 influenza season in Denmark, with A(H1N1)pdm09 dominating at the start of the season and A(H3N2) becoming dominant later in the season (Figure 1). The intensity of the season was medium and an excess mortality was observed in people above 64 years of age in weeks 5–7 2019.

Data are based on subtyping results of all influenza-positive surveillance samples submitted to the National Influenza Centre, Statens Serum Institut, Denmark.

## Case description

In late May 2019, surveillance samples that were not included in the running real-time screening were subtyped in a follow-up round at the NIC. One sample was found to be positive for all gene targets used in the initial multiplex quantitative reverse transcription-polymerase chain reaction (qRT-PCR) detecting all influenza A viruses using the matrix gene, the H1pdm09 gene and N2 gene with similar ct-values, 31.42, 31.62 and 31.59, respectively. The sample was then tested in qRT-PCRs detecting the N1pdm09 and H3 genes with negative results. The sample was from an unvaccinated woman in her 70s with asthma and chronic obstructive pulmonary disease (COPD) who was admitted to a Danish hospital on 11 April 2019 with signs of lower respiratory tract infection. The patient had travelled to Croatia, Bosnia and Herzegovina, and Montenegro between 29 March and 5 April 2019. She flew to Croatia and travelled through the three countries on an arranged tour. After returning, she developed symptoms of lower respiratory tract infection on 7 April and was hospitalised 4 days later. Influenza A virus infection was confirmed. Her symptoms were compatible with influenza. She was discharged on 13 April and has since recovered fully.

The patient’s travel companion remained well during and after the trip. None of the other tourists on the tour developed symptoms during the trip. No information regarding their health status after the trip was available. No contacts of the patient had any symptoms of influenza after her return from her travel.

## Virus characterisation

After real-time RT-PCR detection and confirmation of the initial findings, the sample was further run in conventional RT-PCRs detecting the full genes of seasonal H1pdm09, N1pdm09, H3, N2 and matrix, respectively, as well as whole genome one-tube RT-PCR [1,2]. The sample was still positive only for H1pdm09 and N2 genes. The PCR products of the single genes were sequenced by Sanger sequencing, as well as, together with the whole genome sequencing (WGS) products by next generation sequencing.

**Figure 2:**
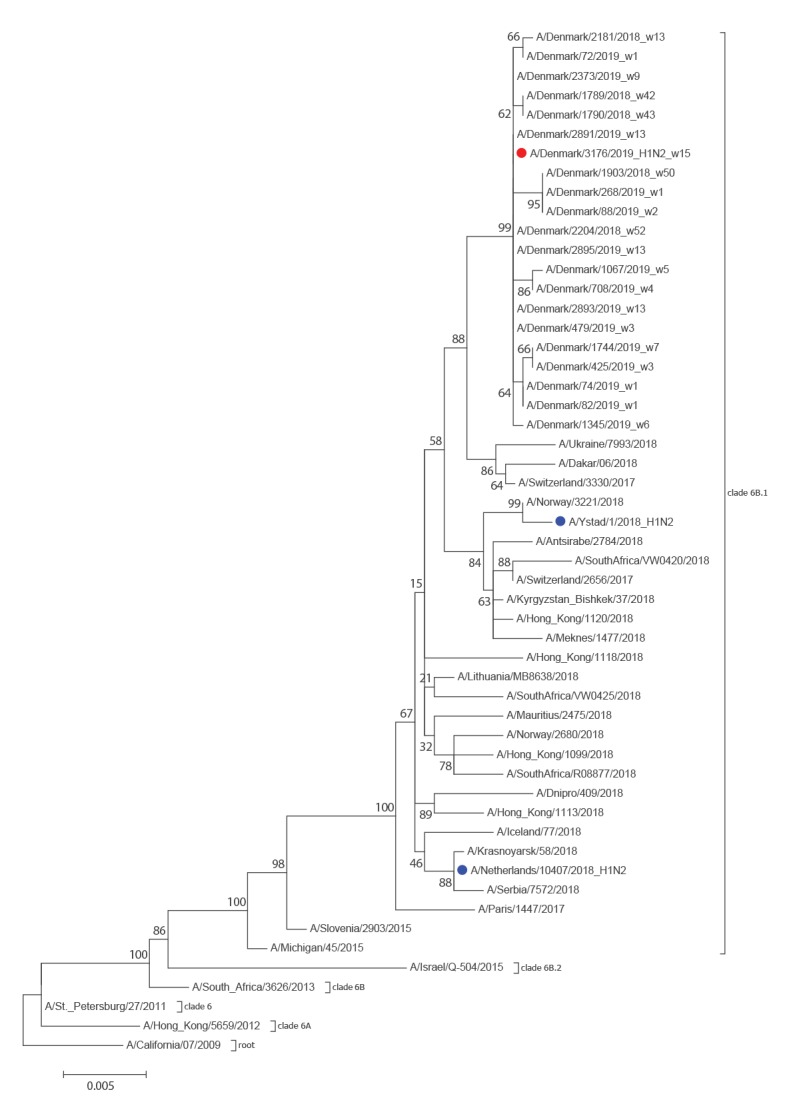
Phylogeny of the haemagglutinin gene of seasonal A(H1N1)pdm09 viruses, Denmark, influenza season 2018/19

Investigation of the haemagglutinin (HA) gene by phylogeny and BLAST in Global Initiative on Sharing All Influenza Data (GISAID) (Supplement S1) and National Center for Biotechnology Information (NCBI) databases showed clustering with seasonal A(H1N1)pdm09 viruses (weeks 40–20 2018) and the European Centre for Disease Prevention and Control (ECDC) reference A(H1N1)pdm09 viruses for the current season [3]. The homology to the first 500 BLAST hits, all of which were seasonal A(H1N1)pdm09 viruses, ranged between 99.2% and 100%. Based on the HA gene sequence, it belonged to subclade 6B.1 (Figure 2) with the following mutations: S74R, I295V, S164T, S183P, N129D, N260D and T185I. All of the mutations are also present in the other contemporary circulating A(H1N1)pdm09 viruses.

The influenza A/Denmark/3176/2019 (H1N2) virus is indicated with a red dot. The analysis includes reference A(H1N1)pdm09 viruses provided by the European Centre for Disease Prevention and Control for seasonal analysis and contemporary Danish isolates. The A(H1N2) reassortants from Sweden and the Netherlands are also included and indicated with blue dots. The phylogenetic tree was constructed by the maximum likelihood method in MEGA6 with bootstrap replicates of 1,000 [15].

**Figure 3:**
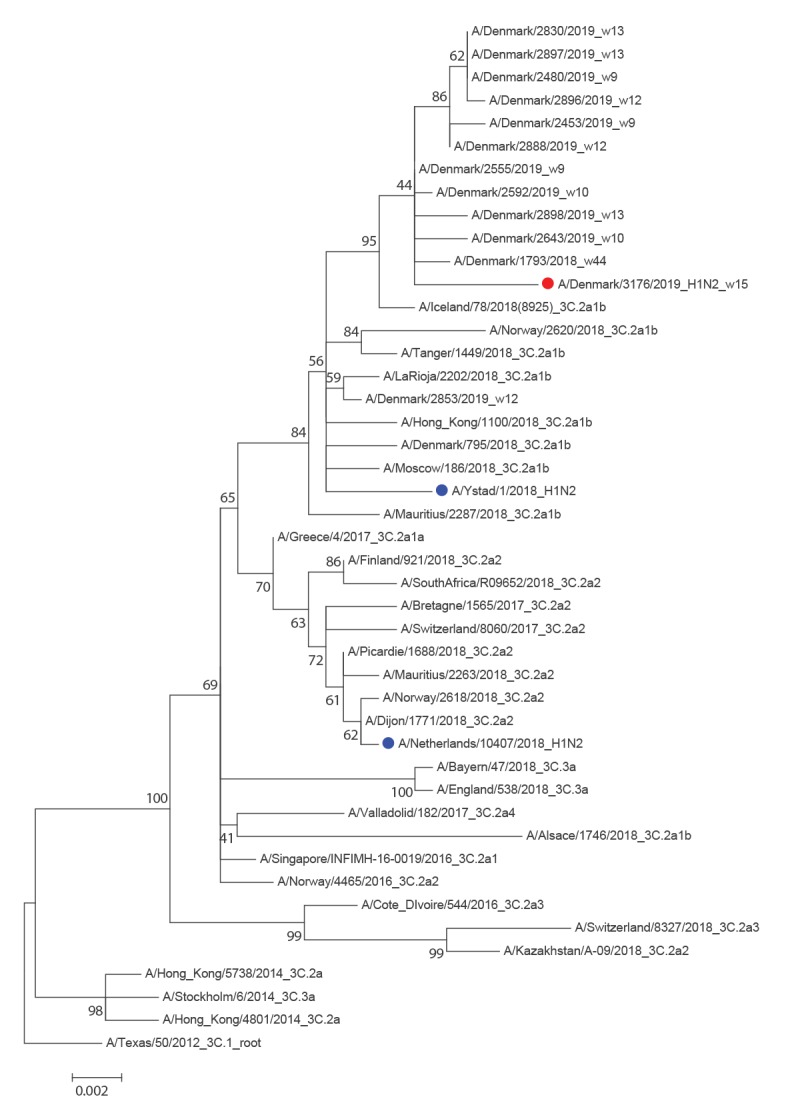
Phylogeny of the neuraminidase gene of seasonal A(H3N2) viruses, Denmark, influenza season 2018/19

The neuraminidase (NA) gene was similar to seasonal A(H3N2) viruses attributed to subclade 3C.2a1b (Figure 3). The homology to the first 500 BLAST hits, all of which were seasonal A(H3N2) viruses, ranged between 98.75% and 99.51%. There were no known antiviral resistance mutations against NA inhibitors.

The influenza A/Denmark/3176/2019 (H1N2) virus is indicated with a red dot. The analysis includes reference A(H3N2) viruses provided by the European Centre for Disease Prevention and Control for seasonal analysis and contemporary Danish isolates. The reference viruses have clade designation after the isolate name, the clade designation is based on haemagglutinin phylogeny. The A(H1N2) reassortants from Sweden and the Netherlands are also included and indicated with blue dots. The phylogenetic tree was constructed by the maximum likelihood method in MEGA6 with bootstrap replicates of 1,000 [15].

The remaining six gene segments were analysed by a BLAST search in GISAID and NCBI, and all showed highest identities with the corresponding gene segments of current circulating human A(H1N1)pdm09 viruses. The matrix gene harboured the S31N mutation inferring antiviral resistance against amantadine as other characterised viruses in circulation.

There was no evidence of mixed infection of the two influenza A virus subtypes H1N1pdm09 and H3N2, as the sample was negative for both the H3 gene from A(H3N2) and the N1pdm09 gene from A(H1N1)pmd09 in both real-time and conventional RT-PCRs, and all six internal gene sequences (MP, NS, NP, PA, PB1 and PB2) matched those of A(H1N1)pdm09 viruses.

The virus is named A/Denmark/3176/2019 and has a GISAID isolate number of EPI_ISL_356893.

A virus isolate of the A(H1N2) virus was successfully obtained in MDCK-SIAT cells, but not MDCK cells. Antigenic characterisation was performed by hemagglutination inhibition (HAI) test using reference ferret antiserum against vaccine viruses provided by the World Health Organization (WHO) Collaborating Centre for Reference and Research on Influenza laboratory at the Francis Crick Institute, United Kingdom. Red blood cells from guinea pigs were used for testing and a cut-off of < 20 in HAI titre for evaluation of test results. The A(H1N2) virus had HAI titres comparable to the vaccine reference virus A/Michigan/45/2015 (H1N1)pdm09 indicating antigenic similarity to this virus (Table). Minor reactions were observed against A(H3N2) antisera, which may be a result of cross-reactivity to the NA protein. Antiviral susceptibility testing of the virus was performed using neuraminidase inhibition test with oseltamivir and zanamivir, and normal inhibition was detected.

**Table:** Antigenic characterisation results of seasonal reassortant A/Denmark/3176/2019 virus based on hemagglutination inhibition (HAI) tests, Denmark, April 2019

Influenza virus	Strain name	Ferret antisera				
		A/Michigan/45/2015 (H1N1)pdm09	A/California/07/2009 (H1N1)pdm09	A/Singapore/INFIMH-16–0019/2016 (H3N2)	A/Switzerland/8060/2017 (TC) (H3N2)	A/Switzerland/8060/2018 (egg) (H3N2)
Reference viruses	A/Michigan/45/2015 (H1N1)pdm09	2,560	320	NA	40	NA
	A/California/07/2009 (H1N1)pdm09	320	1,280	NA	80	NA
	A/Singapore/INFIMH-16–0019/2016 (H3N2)	20	NA	1,280	80	120
	A/Switzerland/8060/2017 (TC) (H3N2)	< 20	NA	160	320	160
A(H1N2) reassortant	A/Denmark/3176/2019	2,560	320	40	40	20

NA: not applicable; TC: tissue culture.

HAI titres are indicated for reference viruses and reassortant influenza A subtype H1N2 virus A/Denmark/3176/2019 tested against a panel of ferret antisera against reference viruses.

## Follow up

This was the only reassortant virus detected among more than 3,000 influenza type A viruses that were HA and NA subtyped, and ca 300 viruses characterised by WGS from Denmark week 40 2018 to week 20 2019. The case was notified to the WHO according to the International Health regulations (IHR), and also to the ECDC and the European Commission via the Early Warning and Response System (EWRS). The risk of further transmission of the reassortant was considered low and no further public health measures were taken.

## Discussion

Human seasonal reassortant A(H1N2) influenza viruses harbouring the HA gene from an A(H1N1)pdm09 virus and the NA gene from an A(H3N2) virus were detected in one case in Sweden in December 2018 and one case in the Netherlands during the 2017/18 influenza season [4,5]. The gene constellation of the Danish A(H1N2) reassortant virus was similar to that found in Sweden, with seven genes (HA, MP, NS, NP, PA, PB1 and PB2) of A(H1N1)pdm09 virus origin and only the NA gene originating from seasonal A(H3N2) virus. Whether this gene constellation is favourable for reassortment events is unknown and will require further investigations. The A(H1N2) virus detected in the Netherlands had another gene constellation with HA and NS from the A(H1N1)pdm09 virus and the other genes acquired from the A(H3N2) virus [5].

These recent reassortment events of seasonal influenza A viruses emphasise the importance of using detection assays that cross-combine targets for HA and NA genes of the A(H1N1)pdm09 and A(H3N2) subtypes, respectively. If detection assay algorithms do not take this into account, other means to detect reassortment events should be considered, e.g. Sanger sequencing of HA and NA genes or WGS. Besides the European cases, an A(H1N2) reassortant where the HA gene was from the former seasonal A(H1N1) virus and the other genes from seasonal A(H3N2) virus was detected in China in 1988/89. Circulation of A(H1N2) viruses genetically similar to the Chinese cases was observed globally in humans in 2000 to 2003 [6]. Dual infections with influenza A subtypes are not unusual [7,8], and pose a risk for reassortment events and the occurrence of new viruses. A special concern is the risk of reassortment events between seasonal and zoonotic viruses, which could result in viruses with pandemic potential [9]. It is important that new viruses are detected and their potential for virulence and transmission evaluated to provide proper risk assessments. It is known that the seasonal influenza A virus subtypes (H1N1pdm09 and H3N2) have different characteristics regarding target groups, e.g. A(H3N2) viruses have a higher impact on people above 64 years of age whereas A(H1N1)pdm09 viruses have a higher illness rate in adults 18–64 years of age [10]. New reassortant viruses with a mix of genes from the seasonal viruses might change target group, virulence and transmission potential.

As the gene constellation of the reported reassortant A(H1N2) virus was from seasonal influenza A subtype H1N1pdm09 and H3N2 viruses, it is anticipated that immunity in the population exists from seasonal vaccination and natural infections with seasonal A(H1N1)pdm09 and A(H3N2) viruses. This was further confirmed by the results from the HAI test, which indicated that the reassortant virus had similar antigenic characteristics as the vaccine virus A/Michigan/45/2015. Vaccine effectiveness (VE) studies for the 2018/19 influenza season included reports on substantial VE for A(H1N1)pdm09 viruses [11-13].

Influenza has an incubation time between 1 and 4 days [14]. With development of symptoms 2 days after her return from an 8-day trip to Croatia, Bosnia and Herzegovina, and Montenegro, it is most likely that the patient was infected either during her trip or during the travel back to Denmark. Both influenza A virus subtypes H1N1pdm09 and H3N2 circulated during the 2018/19 influenza season in most of Europe, including Denmark and the countries the patient had visited. In seasons where both influenza A virus subtypes H1N1pdm09 and H3N2 circulate, there is a risk of co-infection with both subtypes and thereby reassortment events.

In conclusion, reassortment events of seasonal influenza A subtype H1N1pdm09 and H3N2 viruses occur. It is important that national reference laboratories are able to detect new reassortant viruses and that the potential public health impact of reassortant viruses is assessed.

## *Authors’ correction

An additional affiliation has been added for Anders Koch: Department of Infectious Diseases, Rigshospitalet University Hospital, Copenhagen, Denmark. This correction was made on 17 July 2019 upon request of the authors.

## Supplementary Data

335000 Supplement S1
