# Authors’ correction for Euro Surveill. 2019;24(27)

**DOI:** 10.2807/1560-7917.ES.2019.24.29.1907181

**Published:** 2019-07-18

**Authors:** Eurosurveillance editorial team

**Affiliations:** 1 European Centre for Disease Prevention and Control (ECDC), Stockholm, Sweden

For the article ‘A case of reassortant seasonal influenza A(H1N2) virus, Denmark, April 2019’ by R Trebbien et al. published on 4 July 2019, an additional affiliation has been added for Anders Koch: Department of Infectious Diseases, Rigshospitalet University Hospital, Copenhagen, Denmark. This correction was made on 17 July 2019 upon request of the authors.

