# Supplementary material for: A case of reassortant seasonal influenza A(H1N2) virus, Denmark, April 2019
Source: Euro Surveill. 2019 Jul 4;24(27):1900406. doi: 10.2807/1560-7917.ES.2019.24.27.1900406 (PMC6628757; doi:10.2807/1560-7917.ES.2019.24.27.1900406)
Supplement: Supplement S1 [file 1900406_TREBBIEN_H1N2_SupplementS1.pdf]

Supplement S1

This supplementary material is hosted by Eurosurveillance as supporting information alongside the article, 'A case of reassortant seasonal influenza A(H1N2) virus, Denmark, April 2019' on behalf of the authors who remain responsible for the accuracy and appropriateness of the content. The same standards for ethics, copyright, attributions and permissions as for the article apply. Eurosurveillance is not responsible for the maintenance of any links or email addresses provided therein.

We acknowledge the authors, originating and submitting laboratories of the sequences from GISAID's EpiFlu™ Database on which this research is based. The list is detailed below.

All submitters of data may be contacted directly via the GISAID website ***www.gisaid.org***

Segment ID	Segment	Country	Collection date	Isolate name	Originating Lab	Submitting Lab	Authors
EPI1142011	HA	France	2017-Oct-20	A/Paris/1447/2017	Institut Pasteur	Crick Worldwide Influenza Centre	
EPI1310110	HA	South Africa	2018-Jun-11	A/SouthAfrica/VW0425/2018	National Institute for Communicable Disease	Crick Worldwide Influenza Centre	
EPI1277090	HA	South Africa	2018-Jun-13	A/SouthAfrica/VW0420/2018	National Institute for Communicable Disease	Crick Worldwide Influenza Centre	
EPI1277080	HA	South Africa	2018-Jun-14	A/SouthAfrica/R08877/2018	National Institute for Communicable Disease	Crick Worldwide Influenza Centre	
EPI697729	HA	Israel	2015-Dec-15	A/Israel/Q-504/2015	Central Virology Laboratory Israel (NIC)	Crick Worldwide Influenza Centre	
EPI1255238	HA	Norway	2018-Apr-16	A/Norway/2680/2018	WHO National Influenza Centre	Crick Worldwide Influenza Centre	
EPI1271993	HA	Mauritius	2018-Jul-04	A/Mauritius/2475/2018	Central Health Laboratory	Crick Worldwide Influenza Centre	
EPI1277064	HA	Madagascar	2018-Jun-18	A/Antsirabe/2784/2018	Institut Pasteur de Madagascar	Crick Worldwide Influenza Centre	
EPI1271957	HA	Hong Kong (SAR)	2018-Jun-18	A/Hong Kong/1120/2018	Government Virus Unit	Crick Worldwide Influenza Centre	
EPI1271953	HA	Hong Kong (SAR)	2018-Jun-17	A/Hong Kong/1118/2018	Government Virus Unit	Crick Worldwide Influenza Centre	
EPI1271943	HA	Hong Kong (SAR)	2018-Jun-15	A/Hong Kong/1113/2018	Government Virus Unit	Crick Worldwide Influenza Centre	
EPI1271937	HA	Hong Kong (SAR)	2018-Jun-13	A/Hong Kong/1099/2018	Government Virus Unit	Crick Worldwide Influenza Centre	
EPI1274844	HA	Serbia	2018-Apr-03	A/Serbia/7572/2018	Institute of Immunology and Virology Torlak	Crick Worldwide Influenza Centre	
EPI1258963	HA	Ukraine	2018-Apr-03	A/Dnipro/409/2018	Institute of Epidemiology and Infectious Diseases AMS of Ukraine	Crick Worldwide Influenza Centre	
EPI1252718	HA	Morocco	2018-Mar-15	A/Meknes/1477/2018	Institut National d'Hygiène	Crick Worldwide Influenza Centre	
EPI687827	HA	Slovenia	2015-Oct-26	A/Slovenia/2903/2015	Laboratory for Virology, National Institute of Public Health	Crick Worldwide Influenza Centre	
EPI1262205	HA	Iceland	2018-Apr-12	A/Iceland/77/2018	Landspitali - University Hospital	Crick Worldwide Influenza Centre	
EPI1271989	HA	Lithuania	2018-Mar-14	A/Lithuania/MB8638/2018	Lithuanian AIDS Center Laboratory	Crick Worldwide Influenza Centre	
EPI1310066	HA	Senegal	2018-Jun-26	A/Dakar/06/2018	Institut Pasteur de Dakar	Crick Worldwide Influenza Centre	
EPI1153822	HA	Switzerland	2017-Dec-20	A/Switzerland/3330/2017	Hopital Cantonal Universitaire de Geneves	Crick Worldwide Influenza Centre	
EPI1153818	HA	Switzerland	2017-Dec-21	A/Switzerland/2656/2017	Hopital Cantonal Universitaire de Geneves	Crick Worldwide Influenza Centre	
EPI1260044	HA	Ukraine	2018-Apr-04	A/Ukraine/7993/2018	Ministry of Health of Ukraine	Crick Worldwide Influenza Centre	
EPI1274848	HA	Russian Federation	2018-May-03	A/Krasnoyarsk/58/2018	State Research Center of Virology and Biotechnology (VECTOR)	Crick Worldwide Influenza Centre	
EPI1274808	HA	Kyrgyzstan	2018-Apr-10	A/Kyrgyzstan Bishkek/37/2018	National Virology Laboratory, Center Microbiological Investigations	Crick Worldwide Influenza Centre	
EPI1276981	HA	Norway	2018-Jul-24	A/Norway/3221/2018	Drammen Hospital / Vestreviken HF, Department for Medical Microbiology section Drammen	Norwegian Institute of Public Health	Bragstad, K; Dudman, SG; Waalen, K; Hungnes, O
EPI319527	HA	Russian Federation	2011-Feb-14	A/St. Petersburg/27/2011	WHO National Influenza Centre Russian Federation	National Institute for Medical Research	
EPI466626	HA	South Africa	2013-Jun-06	A/South Africa/3626/2013	National Institute for Communicable Disease	National Institute for Medical Research	
EPI390473	HA	Hong Kong (SAR)	2012-May-21	A/Hong Kong/5659/2012	Government Virus Unit	National Institute for Medical Research	
EPI685579	HA	United States	2015-Sep-07	A/Michigan/45/2015	Michigan Department of Community Health	Centers for Disease Control and Prevention	
EPI176620	HA	United States	2009-Apr-09	A/California/07/2009	Naval Health Research Center	Centers for Disease Control and Prevention	
EPI1201795	HA	Netherlands	2018-Mar-05	A/Netherlands/10407/2018	National Institute for Public Health and the Environment (RIVM)	Erasmus Medical Center	Meijer, A.; van den Brink, S.; Bestebroer, T.; Fouchier, R.
EPI1345921	HA	Sweden	2018-Dec-26	A/Ystad/1/2018		Public Health Agency of Sweden	
EPI1255199	NA	France	2018-Apr-04	A/Picardie/1688/2018	Institut Pasteur	Crick Worldwide Influenza Centre	
EPI1255155	NA	France	2018-Apr-05	A/Alsace/1746/2018	Institut Pasteur	Crick Worldwide Influenza Centre	
EPI1145233	NA	France	2017-Nov-27	A/Bretagne/1565/2017	Institut Pasteur	Crick Worldwide Influenza Centre	
EPI1252525	NA	United Kingdom	2018-Feb-26	A/England/538/2018	Microbiology Services Colindale, Public Health England	Crick Worldwide Influenza Centre	
EPI1310137	NA	South Africa	2018-Jun-27	A/SouthAfrica/R09652/2018	National Institute for Communicable Disease	Crick Worldwide Influenza Centre	
EPI1252566	NA	Norway	2018-Apr-16	A/Norway/2620/2018	WHO National Influenza Centre	Crick Worldwide Influenza Centre	
EPI868820	NA	Norway	2016-Nov-07	A/Norway/4465/2016	WHO National Influenza Centre	Crick Worldwide Influenza Centre	
EPI1274877	NA	Mauritius	2018-May-02	A/Mauritius/2263/2018	Central Health Laboratory	Crick Worldwide Influenza Centre	
EPI1270813	NA	Mauritius	2018-May-10	A/Mauritius/2287/2018	Central Health Laboratory	Crick Worldwide Influenza Centre	
EPI1270785	NA	Finland	2018-Apr-16	A/Finland/921/2018	National Institute for Health and Welfare	Crick Worldwide Influenza Centre	
EPI1270791	NA	Hong Kong (SAR)	2018-Jun-12	A/Hong Kong/1100/2018	Government Virus Unit	Crick Worldwide Influenza Centre	
EPI1252576	NA	Morocco	2018-Mar-20	A/Tanger/1449/2018	Institut National d'Hygiène	Crick Worldwide Influenza Centre	
EPI967220	NA	Greece	2017-Jan-02	A/Greece/4/2017	Aristotelian University of Thessaloniki	Crick Worldwide Influenza Centre	
EPI1260053	NA	Iceland	2018-Apr-12	A/Iceland/78/2018(8925)	Landspitali - University Hospital	Crick Worldwide Influenza Centre	
EPI1154885	NA	Spain	2017-Oct-24	A/Valladolid/182/2017	Universidad de Valladolid	Crick Worldwide Influenza Centre	
EPI1255213	NA	Switzerland	2018-Apr-03	A/Switzerland/8327/2018	Hopital Cantonal Universitaire de Geneves	Crick Worldwide Influenza Centre	
EPI1201231	NA	Switzerland	2017-Dec-21	A/Switzerland/8060/2017	Hopital Cantonal Universitaire de Geneves	Crick Worldwide Influenza Centre	
EPI1277125	NA	Kazakhstan	2018-Apr-04	A/Kazakhstan/A-09/2018	National Reference Laboratory	Crick Worldwide Influenza Centre	
EPI1270821	NA	Russian Federation	2018-May-02	A/Moscow/186/2018	Ivanovsky Research Institute of Virology RAMS	Crick Worldwide Influenza Centre	
EPI781597	NA	Cote d'Ivoire	2016-Apr-06	A/Cote D'Ivoire/544/2016	Pasteur Institut of Côte d'Ivoire	Crick Worldwide Influenza Centre	
EPI1256087	NA	Spain	2018-Apr-02	A/LaRioja/2202/2018	Instituto de Salud Carlos III	Crick Worldwide Influenza Centre	
EPI1243179	NA	Germany	2018-Apr-06	A/Bayern/47/2018	Robert Koch Institute Nationales Referenzzentrum für Influenza	Crick Worldwide Influenza Centre	
EPI1277103	NA	Denmark	2018-Apr-16	A/Denmark/795/2018	Statens Serum Institute	Crick Worldwide Influenza Centre	
EPI530678	NA	Sweden	2014-Feb-06	A/Stockholm/6/2014	Public Health Agency of Sweden	National Institute for Medical Research	
EPI539807	NA	Hong Kong (SAR)	2014-Apr-30	A/Hong Kong/5738/2014	Government Virus Unit	National Institute for Medical Research	
EPI539577	NA	Hong Kong (SAR)	2014-Feb-26	A/Hong Kong/4801/2014	Government Virus Unit	National Institute for Medical Research	

EPI1047603	NA	Singapore	2016-Jun-14	A/Singapore/INFIMH-16-0019/2016	WHO Collaborating Centre for Reference and Research on Influenza	Centers for Disease Control and Prevention	
EPI391246	NA	United States	2012-Apr-15	A/Texas/50/2012	Texas Department of State Health Services-Laboratory Services	Centers for Disease Control and Prevention	
EPI1345920	NA	Sweden	2018-Dec-26	A/Ystad/1/2018		Public Health Agency of Sweden	
EPI1252564	NA	Norway	2018-Apr-16	A/Norway/2618/2018	WHO National Influenza Centre	Crick Worldwide Influenza Centre	
EPI1252807	NA	France	2018-Apr-10	A/Dijon/1771/2018		Institut Pasteur	
EPI1201794	NA	Netherlands	2018-Mar-05	A/Netherlands/10407/2018	National Institute for Public Health and the Environment (RIVM)	Erasmus Medical Center	Meijer, A.; van den Brink, S.; Bestebroer, T.; Fouchier, R.
